# Clinical effect of catgut implantation at acupoints for allergic rhinitis: study protocol for a randomized controlled trial

**DOI:** 10.1186/1745-6215-14-12

**Published:** 2013-01-10

**Authors:** Xinrong Li, Qinxiu Zhang, Luyun Jiang, Tao Li, Min Liu, Huanxing Liu, Xiaopei Wang, Fubing Zhang

**Affiliations:** 1 Chengdu University of Traditional Chinese Medicine, 610072, Chengdu, Sichuan Province, PR China; 2 Department of Otorhinolaryngology, Head and Neck Surgery of the Teaching Hospital of Chengdu University of Traditional Chinese Medicine, 610072, Chengdu, Sichuan Province, PR China

**Keywords:** Allergic rhinitis, Catgut implantation at acupoints, Randomized controlled trial

## Abstract

**Background:**

Catgut implantation at acupoints has been used in China to treat allergic rhinitis (AR) for a long time. However, its efficacy and safety in the treatment of AR is controversial due to the poor quality of the clinical trial of this therapy. This study aims to identify whether catgut implantation at acupoints is indeed an effective and safe treatment for patients with persistent or intermittent allergic rhinitis (PER or IAR) by comparing with sham catgut implantation treatment.

**Methods and design:**

This study compares real *versus* sham catgut implantation at acupoints in 242 patients with a history of PER or IAR and with a positive skin prick test (SPT). The trial will be conducted in the Teaching Hospital of Chengdu University of Traditional Chinese Medicine. In the study, patients will be randomly assigned by computer-generated randomization list into two groups and assessed prior to treatment. Then, they will receive two sessions of treatments (once per 2 weeks) for 4 consecutive weeks and have a follow-up phase of 12 weeks. The administration of catgut implantation (or sham-control) at acupoints follows the guidelines for clinical research on acupuncture (WHO Regional Publication, Western Pacific Series No.15, 1995), and is performed double-blindly by a well-trained physician in acupuncture. The main outcome measures include the primary and secondary indicators. Primary indicators are subjective symptoms scores evaluated by visual analogue scales (VAS) and Rhinoconjunctivitis Quality of Life Questionnaires (RQLQ). The secondary indicators are the results of laboratory examinations, such as serum allergen-specific IgE, nasal inflammatory cells counts (mast cells, eosinophils, and T cells) and nitric oxide concentration in nasal excretion. The use of anti-allergic medication will also be recorded as one of the secondary indicators. Furthermore, adverse events will be recorded and analyzed. If any participants withdraw from the trial, intention-to-treat analysis (ITT) and per-protocol (PP) analysis will be performed.

**Discussion:**

The important features of this trial include the randomization procedures, large sample, and a standardized protocol of catgut implantation at acupoints. This trial will be the first study with a high evidence level in China in order to assess the efficacy and safety of catgut implantation at acupoints in treatment of AR following a randomized, double-blind sham-controlled method.

**Trial registration:**

Chinese Clinical Trial Registry: ChiCTR-TRC-12002191

## Background

Allergic rhinitis (AR) is an IgE-mediated immune disease triggered by allergens with symptoms of nasal obstruction, itching, watery rhinorrhea, and sneezing. AR has become a major health problem worldwide. It has been estimated by the ARIA (Allergic Rhinitis and its Impact on Asthma) 2008 document that over 600 million patients from all countries, in all ethnic groups, and of all ages suffer from AR [1]. In China, the prevalence of allergic rhinitis has increased to 24.6% in 2007 according to the epidemiological study in 11 major cities in China [2]. Furthermore, there is a well-documented relationship existing between nasal allergy and asthma. The prevalence of asthma in patients with rhinitis could reach up to 40%, and the majority of patients with asthma experience rhinitis symptoms [1].

AR causes an impairment on the quality of life (QOL) of patients and results in significant healthcare costs. As an available and affordable treatment choice, acupuncture has been accepted as a therapeutic option for relieving symptoms of AR in China and other countries [3-7]. Catgut implantation at acupoints is a subtype of acupuncture, which can be embedded in the acupoint by using a special needle. A period of time may be needed for the catgut to be completely absorbed by the tissue. Therapeutic effects can be achieved by continuing stimulation caused by the catgut at the acupoint. Therefore, catgut implantation at acupoint may be effective in treating some chronic disease such as AR.

**Figure 1:**
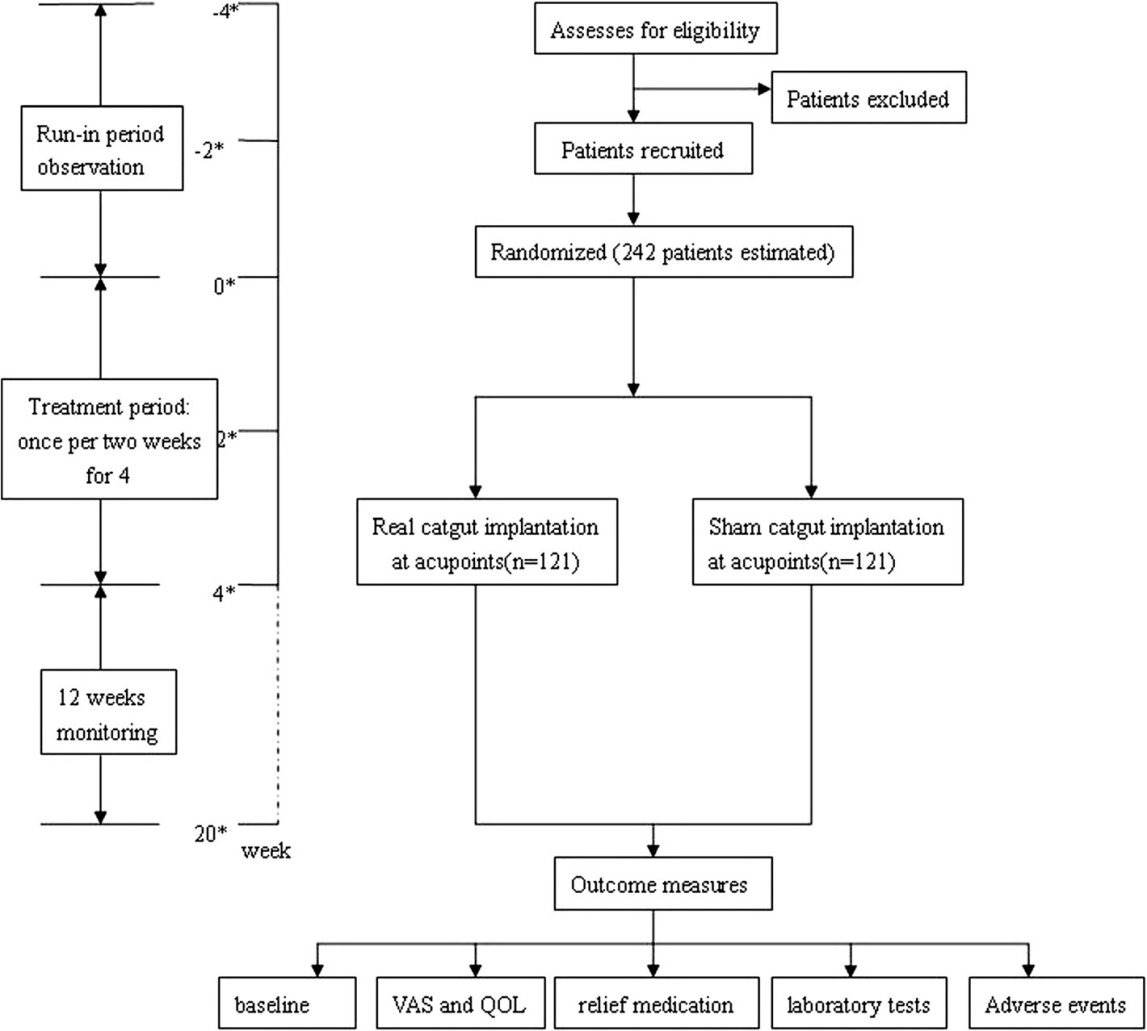
Trial flowchart (*blindly assessed).

Although catgut implantation at acupoint has been used in treating diseases for a few thousand years in China, there have been very few clinical trials which have been strictly designed to verify the efficacy and safety of this treatment for AR [8]. It is indeed necessary to obtain high level of evidences for catgut implantation at acupoints in the treatment of AR (Figure 1).

We have recently conducted a pilot clinical trial to compare the efficiency of catgut implantation at acupoint with triamcinnone acetonide nasal spray in the treatment of AR. The results suggested that therapeutic efficacies were similar between these two groups at the end of the 4-week period of treatment. However, after 3 months of follow-up, the recurrence rate of AR was much lower in the catgut implantation group than in the intranasal glucocorticoid one. Furthermore, symptom-free days were longer in the catgut implantation group.

Based on this pilot study, we wish to design a randomized, double-blind, large-scale clinical trial to compare the effectiveness and safety of real catgut implantation at acupoints with those of the sham one for AR. The design and methodologies of this study has been approved by Sichuan Regional Ethics Review Committee on Traditional Chinese Medicine with an ethics approval number of 2012KL-002. The work reported in this article is registered with an identifier (ChiCTR-TRC-12002191) by Chinese Clinical Trial Registry.

## Methods and design

### Study design

This study will be a double-blind, randomized, sham-controlled trial conducted in the Department of Otorhinolaryngology-head and neck surgery, Teaching Hospital of Chengdu University of T.C.M., China. This study includes the following time points: a run-in period of 4 weeks after screening, a treatment period of 4 weeks, and a follow-up period of 12 weeks. The total study period will be 20 weeks. At the screening visit, medical history especially in relation to AR and other allergic diseases will be asked and recorded by a study physician based on a systemically designed questionnaire. Patients who satisfy the inclusion criteria will be recruited into the run-in period. After randomization, patients will be blindly assigned to either the real catgut implantation at the acupoints group or the sham group (controls). All patients will receive two treatments over a period of 4 weeks. Chlorphenyramine will be used as a rescue medication if the patients present with untolerable AR symptoms. At the same time, patients should complete visual analogue scales (VAS) [9] and Rhinoconjunctivitis Quality of Life Questionnaires (RQLQ) [10,11] before randomization, at the end of treatment, during and after the follow-up period, respectively. Meanwhile, inflammatory cells and mediators in nasal secretions will also be measured at these three visits. Outcome measurements will be assessed by evaluating the efficacy and safety of catgut implantation at acupoints in the treatment of AR.

The design of this study followed the guidelines of the Helsinki Declaration (Version Edinburgh 2000) and was approved by Sichuan Regional Ethics Review Committee on Traditional Chinese Medicine (TCM). Informed consent are to be obtained from all the study patients. Before signing the consent, all patients will be given enough time to decide whether to participate in the trial. Other treatment options instead of catgut implantation at acupoints will be available for those who are unwilling to be recruited in the study.

### Randomization and blinding

Random number of allocation sequence will be generated by computer (using SPSS 15.0 statistical software package). A computer program that allows the patients to be assigned to either the real catgut implantation group or the sham-controlled group in a 1:1 ratio will be used. The random numbers will then be sealed in opaque envelopes. Participants who meet the inclusion criteria will be asked to pickup one of the envelopes. At the same time, the participants’ random numbers and the corresponding randomization information which includes the participant’s name in a pinyin format, the participant’s numerical birthday, and gender will be recorded in a random allocation table in duplicate and sealed in opaque envelopes, respectively. These opaque envelopes should be preserved separately by the principal investigator and the primary sponsor. The envelopes must be kept intact until cease blinding. The administrator who is in charge of random allocation should not participate in enrolling patients. Participants, researchers, and study physicians who interview and recruit patients are blinded to the group assignments. The acupuncturists are responsible for treatment proceedings of both groups, and therefore it would be difficult to blind the acupuncturists during the study. However, they will not communicate with the study patients nor participate in the assessment of study outcomes.

The two randomized groups will be named A or B in the medical records and case report forms which are not directly link to the treatment. All information will be lockedout and saved in a database before statistical analysis is performed. After statistical analysis, the blind exposure will be provided. The two opaque envelopes which contain the random allocation tables should be unsealed by the principal investigator and the primary sponsor simultaneously to clarify which group is the real treatment group or the sham-controlled group.

#### Inclusion criteria

Patients who will be recruited in this study should meet the inclusion criteria including:

(1) aged between 18 and 70 years

(2) not be taking part in any other clinical trials

(3) having no previous experience of catgut implantation at acupoints

(4) provide written informed consent

(5) presenting with typical symptoms of AR, such as rhinorrhoa, sneezing, nasal obstruction, and pruritus. These symptoms should last >1 h on most days. Some patients may have ocular symptoms due to outdoor allergens

(6) with positive skin prick tests performed by trained health professionals

A standardized vaccine (Alutard SQ, ALK- Abelló, Denmark) will be used in the prick test. Oral H1-antihistamines are not permitted before skin test. Skin prick tests should be read at the peak of their reaction by measuring the wheal and the flare approximately 15 min after the performance of the test.

#### Exclusion criteria

Patients with any of the following conditions will be excluded:

(1) pregnant women or women who have been trying to get pregnant in the last 6 months, or women who are lactating

(2) patients who are receiving immune therapy

(3) patients with other allergic diseases, such as bronchial asthma or allergic purpura

(4) patients with nasal polyposis

(5) heterologous protein allergy

(6) patients with other organic disorders, such as AIDS, vascular malformation, hypertension, hematologic diseases, diabetes mellitus, malignant tumor, and mental disorders

#### Withdrawal from the study

Study patients will be allowed or be asked to withdraw from the study if:

(1) they have been included in the trial but would not subject to the assigned treatment for varies reasons at any of the stages of the trial

(2) severe adverse events occur that necessitate withdraw of patients from the trial

(3) they do not fully participate in treatment or follow-up

### Recruitment

#### Hospital recruiting

In China, most of the patients with symptoms of AR will present at the otorhinolaryngology clinics. It is advisable that all physicians in our department should be familiar with the study inclusion and exclusion criteria and refer the appropriate patients to the PI or study physicians. Then the screening evaluations will be conducted and the information of the patients will be recorded. Patients who satisfy the inclusion criteria and are willing to participate in the trial will sign on the written informed consent. Then they will be randomized into either of the study groups and introduced to the acupuncturists.

#### Advertisements

Television and newspaper advertisements will be on display. Printed recruitment posters will also be pasted in the hospital and the university campus.

### Interventions

Participants will receive real or sham catgut implantation at acupoints once per 2 weeks. Each patient will have 4 treatment weeks and 12 weeks of follow-up. The interventions are designed according to the documentary records in ancient books and in recent Traditional Chinese Medicine journals. The protocol is developed in consensus with Chinese acupuncturists and acupuncture experts, and is consistent with both the STRICTA (Standards for Reporting Interventions in Controlled Trials of Acupuncture) guidelines for the performance of acupuncture studies [12] and the guidelines for clinical research on acupuncture (WHO Regional Publication, Western Pacific Series No.15, 1995). In our pilot study, five acupoints were used for catgut implantation and the efficacy was similar between the catgut implantation in the acupoints group and the intranasal glucocorticosteroids group. In this trial, the same acupoints will be chosen in both the real and sham treatments. All the treatments will be performed by the same registered acupuncturists to ensure participant blinding and consistency of treatment.

#### Rationale for the selection of acupoints

**Figure 2:**
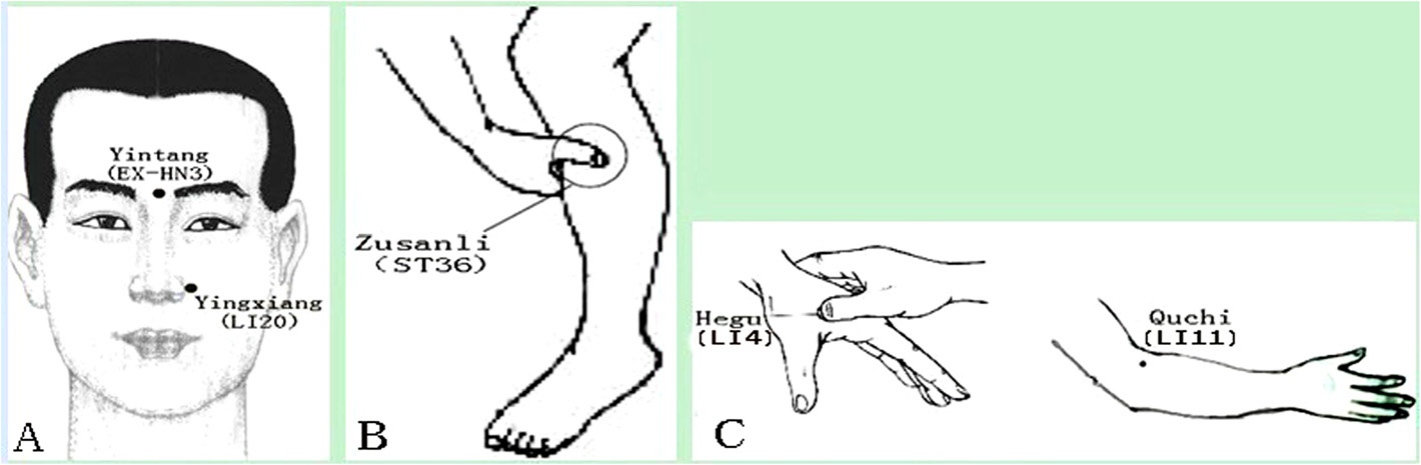
**Locations of acupoints (for both real treatment group and sham**-**controlled group).** Yintang(EX-HN3), yingxiang (LI20), and Zusanli (ST36) are chosen in the first two weeks of treatment. Hegu(LI4) and Quchi (LI11) will be punctured in the second two weeks of treatment.

For the first 2 weeks of treatment, three acupoints are to be used for each patient. They are Yingxiang (LI20), Yintang(EX-HN3), and Hegu(LI4). For the second 2 weeks of treatment, Zusanli(ST36) and Quchi(LI11) will be chosen (Figure 2).

The traditional acupuncture theories state that AR is in close correlation with the Taiyin lung meridian of hand and Yangming large intestine meridian of hand. Therefore, it is reasonable that the acupoints corresponding to the lung meridian and the large intestine meridian would be the most effective acupoints in treating AR. Furthermore, the traditional acupuncture theory maintains that special points convey special therapeutic effects. Thus, the most frequently used specific acupoints for these two meridians would be selected in this trial according to a previous review of ancient and modern literature.

In traditional Chinese medicine, the nose is an orifice with lucid Yang. Yingxiang and Yintang are on the median line of face. Lucid Yang is confluent at these acupoints. These two acupoints plays an important role in clearing nasal passage. In addition, Yingxiang is considered a specific point on Yangming large intestine meridian of hand. Hegu is the source-point of the large intestine meridian. Based on the theory of the lung and the large intestine being interior-exteriorly related, Yingxiang and Hegu are important acupoints for facial features. Yintang is an extraordinary point. The three acupoints could be used together for improving lung air (qi) deficiency syndrome. In our previous trial, it has been suggested that symptoms of AR could be effectively alleviated by embedding catgut at these three acupoints.

Zusanli (ST36) and Quchi(LI11) are sea points of Yangming large intestine meridian of hand and Yangming stomach meridian of foot. The ancient Chinese believed that Yangming is a meridian abundant of blood and Qi and is the acquired foundation. Zusanli and Quchi will be used to fortify the spleen and replenish lung qi. By reinforcing earth to generate metal and by regulating asthenia-cold in the remission stage of AR, recurrence rate of this disease might be reduced.

#### Sham-controlled group

In the control group, the same acupoints are to be punctured with the same acupuncture needles and same length and angle according to standard techniques. The only difference between these two groups is that catgut would not be embedded into the acupoints in the sham-controlled group. Because of mild swollen tissue at acupoints immediately after puncture and lack of previous experiences for this therapy, the patients will not know whether catgut has been embedded into the acupoints. To ensure the blinding manner, acupuncturists are instructed not to communicate with the participants of whole treatment procedures.

#### Standard protocol for catgut implantation at acupoints

Only pre-sterilized disposable needles (9#, Hanjiang Guoxiang Medical Appliance Factory, Yangzhou, China) and catguts (000, Pudong Jinhuan Medical Products Co. Lit, Shanghai, China)are used in the trial. For Yintang (EX-HN3), the needle is to be inserted with a depth of 1.0 cm, downward to the nose in a horizontal direction with respect to the shin. For Yingxiang (IL20), the penetration is 2.0 cm, in an oblique direction along the nasolabial sulcus towards the root of nose with respect to the skin. For Hegu, Zusanli, and Quchi, the penetration is 2.5 cm, in perpendicular direction with respect to the skin (Table 1). Twirling, lifting, and thrusting of the needle do not need to be performed. Both groups will receive one session of catgut implantation every 2 weeks for 4 weeks. Subjects will be advised to lie on a supine position. Pulse rate, blood pressure, and oxygen saturation are to be monitored routinely during the procedure as a precautionary measure. In both groups, pre-injection swabs with 75% alcohol and dry sterile cotton wool are to be used when withdrawing the needles. Before the puncture, it is advised to inject 0.5% Lidocaine of about 0.2 to 0.4 mL for each acupoint to alleviate the pain caused by inserting the needle. After treatment, the skin of the acupoints is prohibited to touch water or any cosmetics for 3 days.

**Table 1:** Acupoints and manipulation for both real and sham catgut implantation group

**Time**	**Acupoints**	**Angle and direction**	**Depth (cm)**
First week of treatment	Yintang(EX-HN3)(unilateral)	Downward to the nose in a horizontal direction with respect to the shin	1.0
	Yingxiang(LI20)(both)	In an oblique direction along the nasolabial sulcus towards the root of nose with respect to the skin	2.0
	Hegu(LI4)(both)	In perpendicular direction with respect to the skin	2.5
Second week of treatment	Zusanli(ST36)	In perpendicular direction with respect to the skin	2.5
	Quchi(IL11) (both)	In perpendicular direction with respect to the skin	2.5

**Figure 3:**
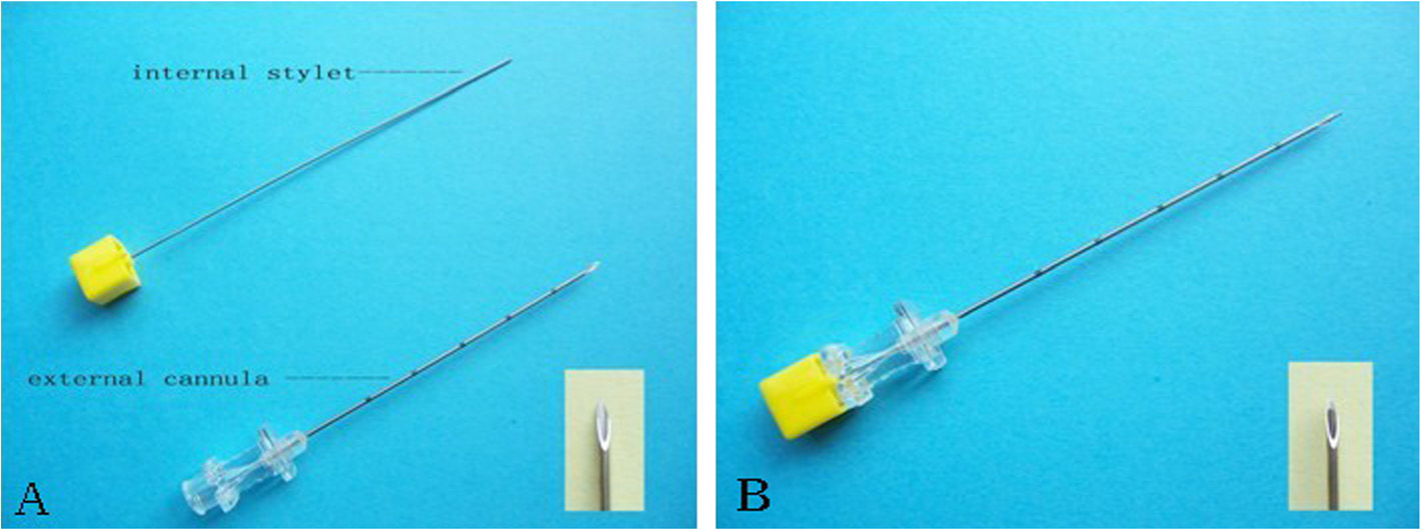
Needle for catgut implantation at acupoints.

The needle used for catgut implantation is similar to a kind of trocar. It is made up of two parts: the internal stylet and the external cannula with sharp pinhead which assures the cannula could puncture into the skin (Figure 3). In real catgut embedding, the internal stylet should be withdrawn from the cannula for about 1.5 cm at first. Then a catgut with the length of 2 to 3 mm will be put into the cannula from the side of pinhead. When the needle has been inserted into the acupoints with the correct depth, the catgut will be pushed into the tissue of the acupoint by the stylet. For the control group, the whole therapeutic procedure is the same as the real group except that the catgut is not present in the cannula. Patients in the control group will receive only the stimulus of needling but not of the catgut implantation.

All real and sham catgut implantation are to be performed by the same acupuncturist consistently throughout the study. The acupuncturists should have undergone at least 8 years of acupuncture training and be qualified TCM doctors.

In both groups, patients are permitted to use chlorphenyramine when needed. The use of chlorphenyramine (dosage, frequency, and period of treatment) will be recorded as relief medicine scores.

### Outcome measurements

#### Primary outcome

The primary outcome measures will be the severity of the AR symptoms assessed by (VAS) [9] and the RQLQ [10,11]. In VAS, the severity of the combination of the nasal symptoms will be subjectively rated by the study patients on the scale. The severity of symptoms can be divided into mild, moderate, or severe based on the total severity VAS score (0 to 10 cm: mild, VAS 0–3; moderate, VAS 3.1-7; severe, VAS 7.1-10). VAS scores will be recorded every 2 weeks during the whole trial. Influence of AR on QOL will be measured by questionnaire every 4 weeks: at baseline, at the end of treatment, during the follow-up period, and at the end of follow-up period (a total of five times). Both the scores of VAS and RQLQ questionnaire will be blindly assessed.

#### Secondary outcome

Secondary outcome measures include relief medication scores, serum allergen-specific IgE, nasal inflammatory cells counts (mast cells, eosinophils, and T cells), the level of nitric oxide concentration in nasal secretion, and side effects.

Patients will be asked to complete diaries in the baseline and every 2 weeks after randomization. In the diaries, they will document the intensity, frequency, length, and symptoms in each AR attack. The patients will also record if H1-blocker (chlorphenyramine) has been taken during the whole treatment periods, including the dosage and time of medication. In addition, adverse events (for example, numbness, hematoma, local infection of the acupuncture sites, headache, fainting, and serious pain) will also be recorded during the treatment and follow-up phases.

### Patient safety

Before randomization, patients will be asked to undergo routine tests of blood, urine, and stool, as well as electrocardiogram (ECG), liver function, hepatitis serology, serum antibody of AIDS, blood glucose, and kidney functions in order to exclude any related serious illness. The results of these tests will allow the assessment of risks associated with catgut implantation at acupoints. If any adverse effect occurs during the trial, patients will contact with the principal investigator as soon as possible to get proper medical treatment.

### Quality control

All acupuncturists are required to receive special training, including the technique for catgut implantation and the way to deal with adverse events. The acupuncturists should also learn how to use the randomization method in order to communicate with patients and to keep them blind to the treatment throughout the trial. Only those who have completed the required training and have passed all necessary examinations will be recruited for this trial. In order to maintain quality of this trial, all outcome assessments will be blinded. In addition, all researchers must understand the purpose and design of this trial. Audits will be conducted regularly on compliance with standard operation procedures every week. Report of audits should be presented to the chief monitor. If patients withdraw from the trial either in the treatment period or in the follow-up phase, the reason should be clarified and the rate should be analyzed in statistics.

### Statistical analysis

In our preliminary study to compare the efficacy of catgut implantation at acupoints with intranasal glucocorticoid, it was suggested that efficiency was 93.75% in the catgut implantation group and 96.88% in the control group. In this study, we anticipate an improvement of no less than 15% after real catgut implantation at acupoints. However, there are no previous data regarding the VAS or RQLQ scores for the treatment of AR with catgut implantation at acupoints. Considering participants recruited in the two groups are in a 1:1 ratio, the following formula was used to estimate sample size [13]:

$$n_{1}=n_{2}={\left[\frac{Z_{\alpha }\sqrt{2\pi_{c}\left(1-\pi_{c}\right)}+Z_{\beta }\sqrt{\pi_{1}\left(1-\pi_{1}\right)+\pi_{2}\left(1-\pi_{2}\right)}}{\pi_{1}-\pi_{2}}\right]}^{2}$$

In the formula: n1, n2 represents the sample size of each group. π1, π2 is the overall rate of each sample, respectively. πc = (π1 + π2)/2, α = 0.05, Z_0.05_ = 1,96,β = 0.10,Z_0.10_ = 1.282. Statistical analysis will be performed using a 5% significance and 90% power, resulting in an estimated 121 patients per group.

To test our hypothesis, the first step is to assess the baseline characteristics of two groups using an unpaired *t* test or *x*^2^ testes. The second step is to compare the efficacy of real catgut implantation with the sham one, including the primary and secondary outcomes using two-sample t test. If there is any case lost in follow-up phase or withdrawn from the trial, the causes of withdrawal should be clarified. The intention-to-treat analysis (ITT) and per-protocol (PP) analysis should be conducted to test whether the result of this trial is credible. For relief medication, within-group as well as between-group comparisons of scores are to be made. Data will be analyzed using the Statistical Package for the Social Sciences software, version 15.0 for Windows (SPSS Inc., Chicago, IL, USA). Data are presented as mean and 95% confidence intervals (CI). The statistical analysis will be performed in a blinded manner by qualified statisticians.

## Discussion

Based on the theory and practice of TCM and acupuncture, it indicates that acupuncture is effective in treating some chronic diseases. Acupuncture has proven to be useful in relieving symptoms of AR. In Western countries, acupuncture has been gradually accepted based on evidence from randomized, double-blind clinical trials in the treatment of AR.

Besides conventional puncture and moxibustion, acupuncture can be performed by such as acupoint application, auricular acupoint pressing, and catgut implantation at acupoints. Catgut implantation at acupoints can extend the sensation of needling because of the persistent stimulus to acupoints by embedding catgut. Furthermore, catgut implantation has an effect which is similar to pricking blood therapy. Hence, it was found to be able to dredge the channels and invigorate the pulse, and regulate qi and blood. Catgut implantation at acupoints has a particular therapeutic effect for some chronic diseases.

In our pilot study, the efficacy and safety of catgut implantation at acupoints were compared with the intranasal glucocorticoid treatment. It has been shown that the clinical efficacy was similar in the two groups and catgut implantation at acupoints were well tolerated by participants. However, the limitations of this pilot study are due to a small sample size (only 64 subjects were enrolled) and it was not performed in a blinded manner. To our knowledge, there has been no other randomized, double-blinded controlled trial of catgut implantation at acupoints for AR [14-16]. In order to obtain more useful clinical evidence for this therapy, we have designed this trial following the principles of good clinical trials.

In the literature, a technique in which needles are inserted shallowly at locations of 1 to 2 cm away from the defined acupoints is a common sham control for clinical study for acupuncture. In our study, the sham treatment is to insert needles at the same acupoints with the same acupuncture manipulation but without catgut embedding. In order to ensure the efficacy and safety of the treatment, acupuncturists should undergo special training and be qualified by having sufficient knowledge, special technique, and good experience in acupuncture. In addition, it is performed in a design of double-blind and randomized manner. All study patients will be informed by potential treatment of either real catgut implantation at acupoints or the sham one. The sham treatment may have unspecific physiologic effects of needling or just have placebo effects. In this study, a short acting antihistamine is permitted as a rescue medication. All patients are required to keep a record of the usage of drugs and relief medication scores will be calculated.

In conclusion, the aim of this study is to investigate the efficacy and safety of catgut implantation at acupoints for treatment of AR. It will be the first time that good clinical evidence of this treatment in AR will be obtained.

### Trial status

The recruitment of patients started June 1,2012, and it is expected that by March 31, 2013 the required sample size will be reached.

## Abbreviations

AR: Allergic rhinitis; IAR: Intermitten allergic rhinitis; ITT: Intention-to-treat analysis; PER: Persistent allergic rhinitis; PP: Per-protocol; RQLQ: Rhinoconjunctivitis Quality of Life Questionnaires; SPT: Skin prick test; TCM: Traditional Chinese Medicine; VAS: Visual analog scales

## Competing interests

The authors declare that they have no competing interests.

## Authors’ contributions

QXZ, XRL, HXL, and ML contributed to the conception and design of the study. XRL drafted the manuscript. All authors contributed to the further writing of the manuscript. All authors read and approved the final manuscript.
